# Supplementary material for: In-hospital real-time prediction of COVID-19 severity regardless of disease phase using electronic health records
Source: PLoS One. 2024 Jan 25;19(1):e0294362. doi: 10.1371/journal.pone.0294362 (PMC10810421; doi:10.1371/journal.pone.0294362)
Supplement: S1 File — (DOCX) [file pone.0294362.s001.docx]

**Supplement methods**

The schema of static models.

The input data for the subsequent models were identical and comprised the following elements: Vital signs, which were condensed to an 8-hour basis per day using statistical measures such as mean, median, maximum, minimum, count, and variance. Additionally, daily medication use, symptoms, laboratory results, and demographic characteristics were also included in the input data.

1. Logistic regression model:

Logistic Regression is a classification algorithm in the machine learning realm, capable of estimating the likelihood of a categorical outcome. We configured a Logistic Regression model utilizing standard hyperparameters, with the sole constant being a specific random seed to ensure the reproducibility of our findings.

2. XG boost model:

XGBoost is an ensemble machine-learning algorithm, grounded in decision-tree principles, that employs a gradient-boosting framework. We established an XGBoost model employing fundamental hyperparameters and specified a consistent random state to ensure the replicability of our results.

3. Random forest model:

Random Forest, an ensemble learning approach, generates a vast array of decision trees during the training process and delivers the class that mirrors the mode of the classes for the purpose of categorization. Utilizing foundational hyperparameters, we devised a Random Forest model with the specific intent of prognosticating the ensuing day's disease severity.

4. Deep learning model:

Our model is comprised of an input layer, three hidden layers, and an output layer, each utilizing a different number of nodes. The model architecture is as follows.

Input Layer: The input layer consists of nodes equal to the number of features in our training data. This layer passes the data directly to the first hidden layer.

First Hidden Layer: This layer contains 512 nodes. The layer utilizes a dropout (20%) regularization technique to prevent overfitting. The activation function employed in this layer is the Rectified Linear Unit (ReLU) function, which introduces non-linearity into the model.

Second Hidden Layer: This layer consists of 1024 nodes. Like the first hidden layer, this layer also uses dropout regularization (20%) the ReLU activation function.

Third Hidden Layer: This layer is made up of 512 nodes. It also applies dropout regularization and the ReLU activation function.

Output Layer: Representing the concluding layer of our model, this layer comprises three nodes. It does not incorporate dropout or an activation function. The quantity of nodes is indicative of the number of categories we aspire to forecast, which in our case are the daily severity levels: mild, moderate, and severe.

The structure of hierarchical transformer

The Transformer model is designed to ingest trend data as its input. In order to facilitate this trend-oriented input, vital signs were consolidated every 8 hours, while laboratory results were consolidated on a daily basis. Daily symptoms were assessed on a day-to-day basis, negating the requirement for further summarization of these particular input variables. The rationale for summarizing vital signs every 8 hours is rooted in the standard practice of Korean hospitals, where vital signs are typically monitored at 8-hour intervals.

Considering the different data frequencies, the hierarchical model consisting of two transformer encoders [1] and deep neural networks. (S Fig 1) For the dynamic data defined by frequently checked data during admission, daily data included laboratory data and daily symptoms. The 8 hour data included vital signs (blood pressure, pulse rate, respiratory rate, temperatures, and pulse oximeter saturation). To summarize the large-scale input to hidden spaces by self-supervised autoencoder, [2] the output of each transformer was connected to the decoder to predict the data itself. The summarized hidden spaces were connected to a hierarchical model to predict daily WHO severity. (S Fig 1)

The transformer could take inputs of variable lengths, which meant that longitudinal admission period data, such as laboratory values and vital signs, could be used to predict an outcome. Given the input length day $k$, the target outcome $t$ occurred after $n$ days, and the input data consisted of *k* consecutive days of input $[t-k-n,t-n]$. Further, the length of daily input was $k$, and that of vital sign data was $3\times k$, given that vital signs were checked every 8 hours. In an earlier prediction where the input length day *k* was longer than the recorded input, the remaining length was padded by a masking value. (S Fig 2)

Hyperparameter tuning: To identify the best model, various hyperparameters were tried, and the hyperparameters used in the training set are described in S Table 1.

S Fig 1. Hierarchical transformer model structure


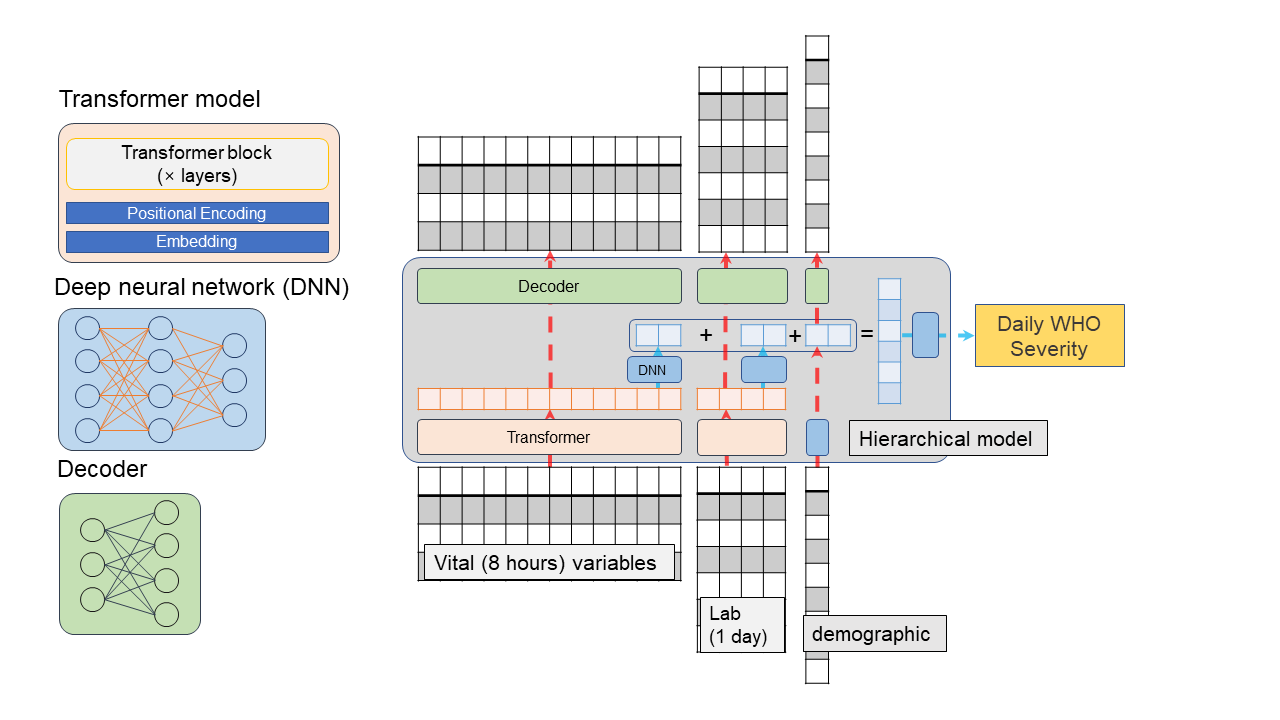


S Fig 2. Schema of the model with different input window sizes and its outcome


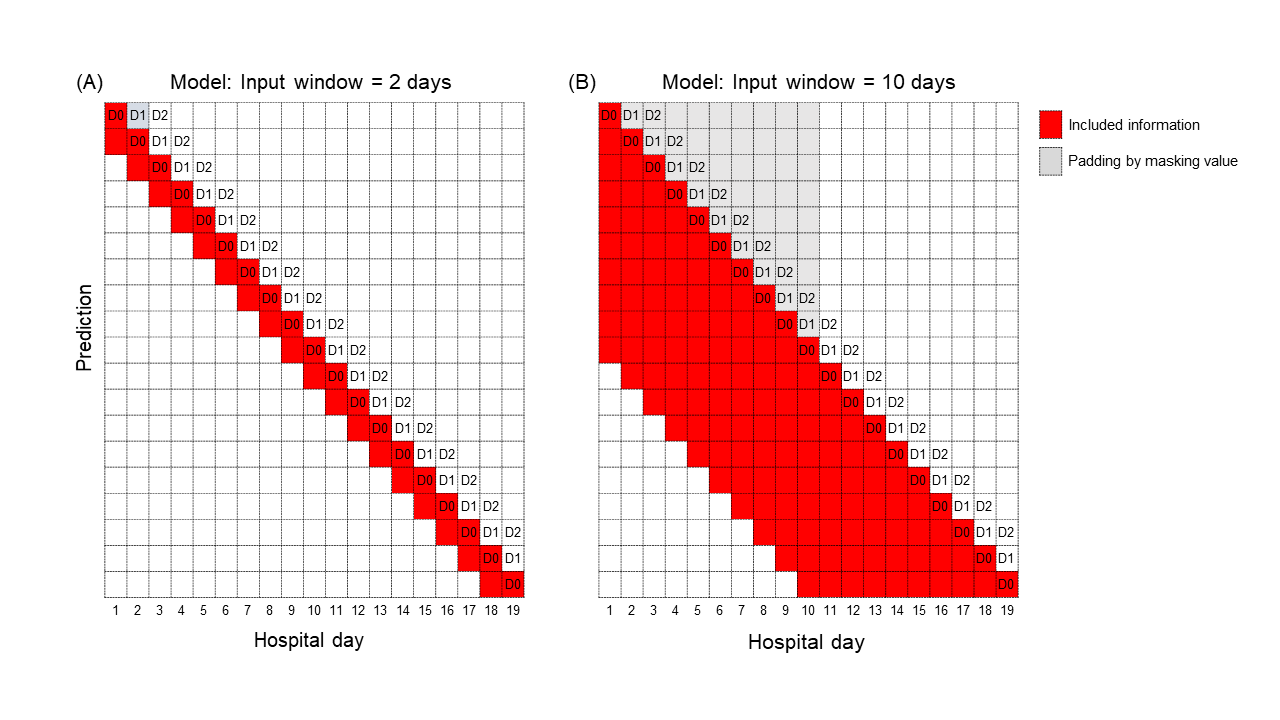
Two models with different input size shows how the data used in each prediction. (A) The model with an input window of 2 days gets the padding only the first time of prediction. (B) The model with an input window of 10 days gets the padding until the 10 hospital days. The larger window model gets more information than that of the smaller window. Although the padding window was imputed by masking value, actual input of the first and second hospital day prediction differed in the two models in aspect of length of input size and time-embedding addition.

D0: outcome event on the last input window date.

D1: outcome event 1 day after the last input window date

D2: outcome event 2 days after the last input window date

S Table 1. Hyperparameters used in training the transformer model

| Overall parameters | | | |
| --- | --- | --- | --- |
| Masking value | [-5,0] | Batch size | 1000 |
| Early stopping | True | Dropout rate | 20% |
| Loss type | [target^1^, total loss^2^] |  |  |
| Transformer parameters | | | |
| Positional embedding number of nodes | [160, 320] | Transformer encoder hidden number | [160, 320] |
| Number of layers | [4, 6, 8] | Number of head | 4 |
| Optimizer: Adam | | | |
| Weight decay | 10^-4^ | Learning rate | $[5\times{10}^{-3}, 5\times{10}^{-4}, 5\times{10}^{-5}, 5\times{10}^{-6}$] |
| Deep neural network: Sequential DNN | | | |
| Number of hidden nodes in each linear layer | [256, 128, 30]^3^ | Construction | [Linear, dropout, ReLU] |

^1^Target loss: cross-entropy loss of target and outcome severity.

^2^Total loss: target loss + reconstruction loss (reconstruction loss was calculated by mean squared error)

^3^The deep neural network used the predefined number of hidden layers.

The hyperparameters in the bracket were used in training to identify the best model.

References

[1] Phuong M, Hutter M. Formal Algorithms for Transformers 2022:1–16.

[2] Le L, Patterson A, White M. Supervised autoencoders: Improving generalization performance with unsupervised regularizers. Adv Neural Inf Process Syst 2018;2018-Decem:107–17.
