# Supplementary figures and images for: In-hospital real-time prediction of COVID-19 severity regardless of disease phase using electronic health records
Source: PLoS One. 2024 Jan 25;19(1):e0294362. doi: 10.1371/journal.pone.0294362 (PMC10810421; doi:10.1371/journal.pone.0294362)

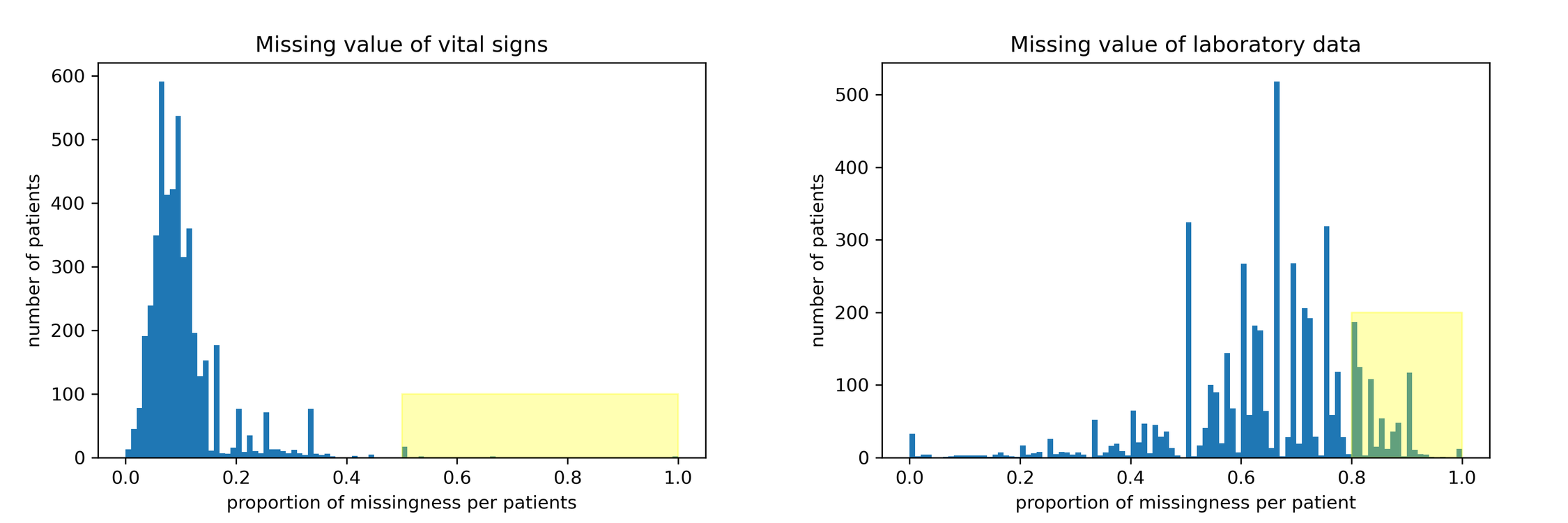

Supplement: S1 Fig — (TIF) [file pone.0294362.s002.tif]

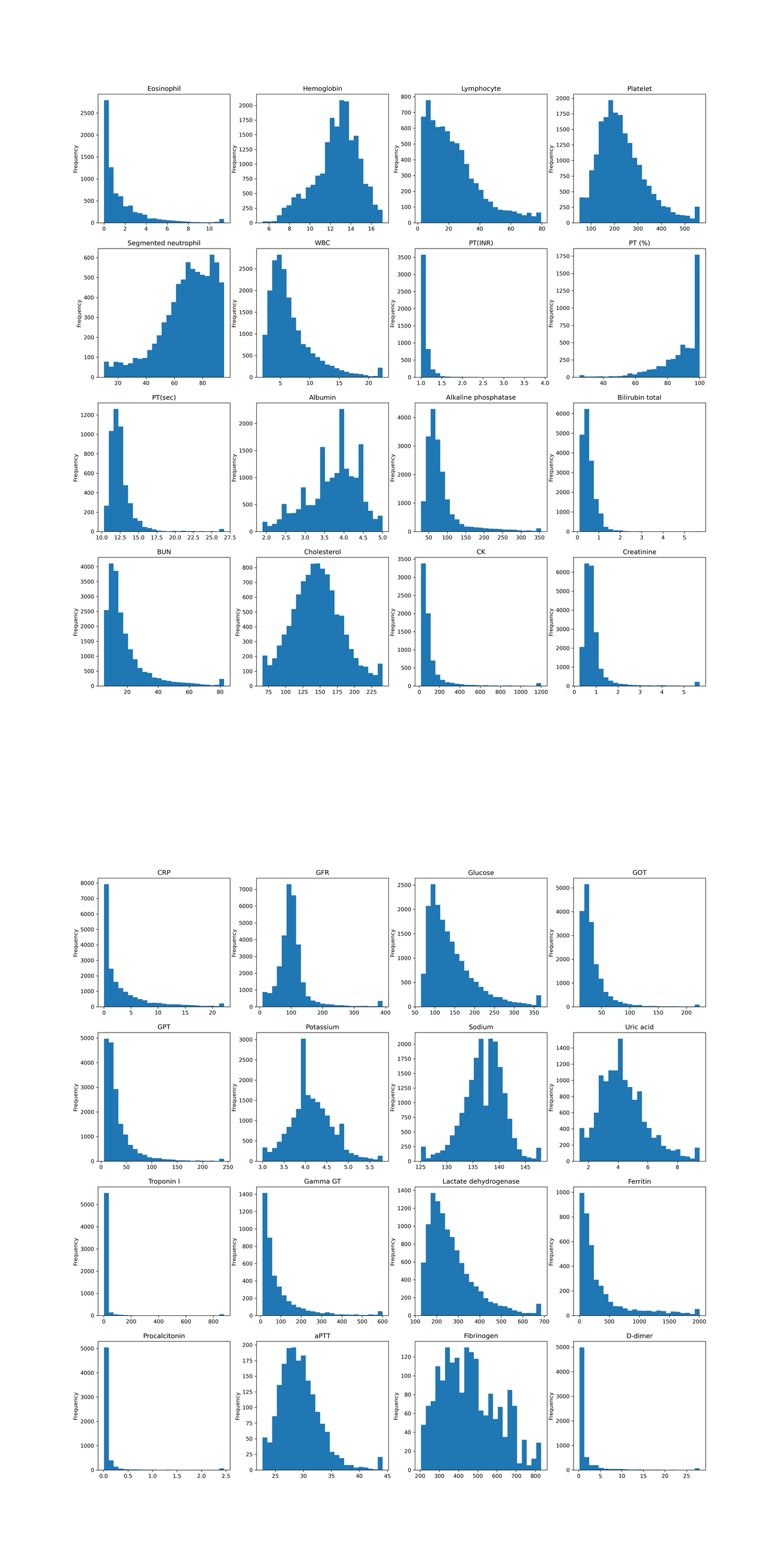

Supplement: S2 Fig — (TIF) [file pone.0294362.s003.tif]

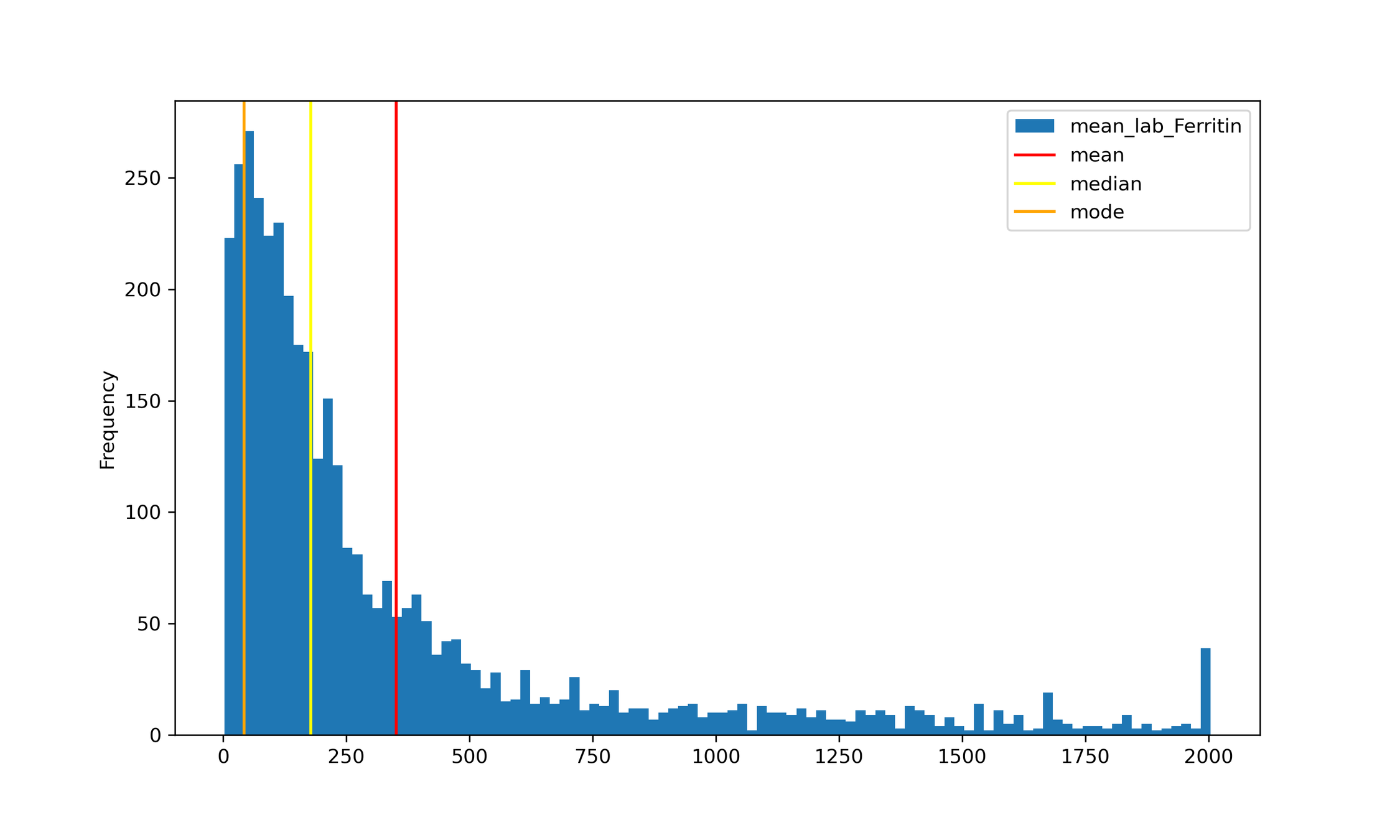

Supplement: S3 Fig — (TIF) [file pone.0294362.s004.tif]

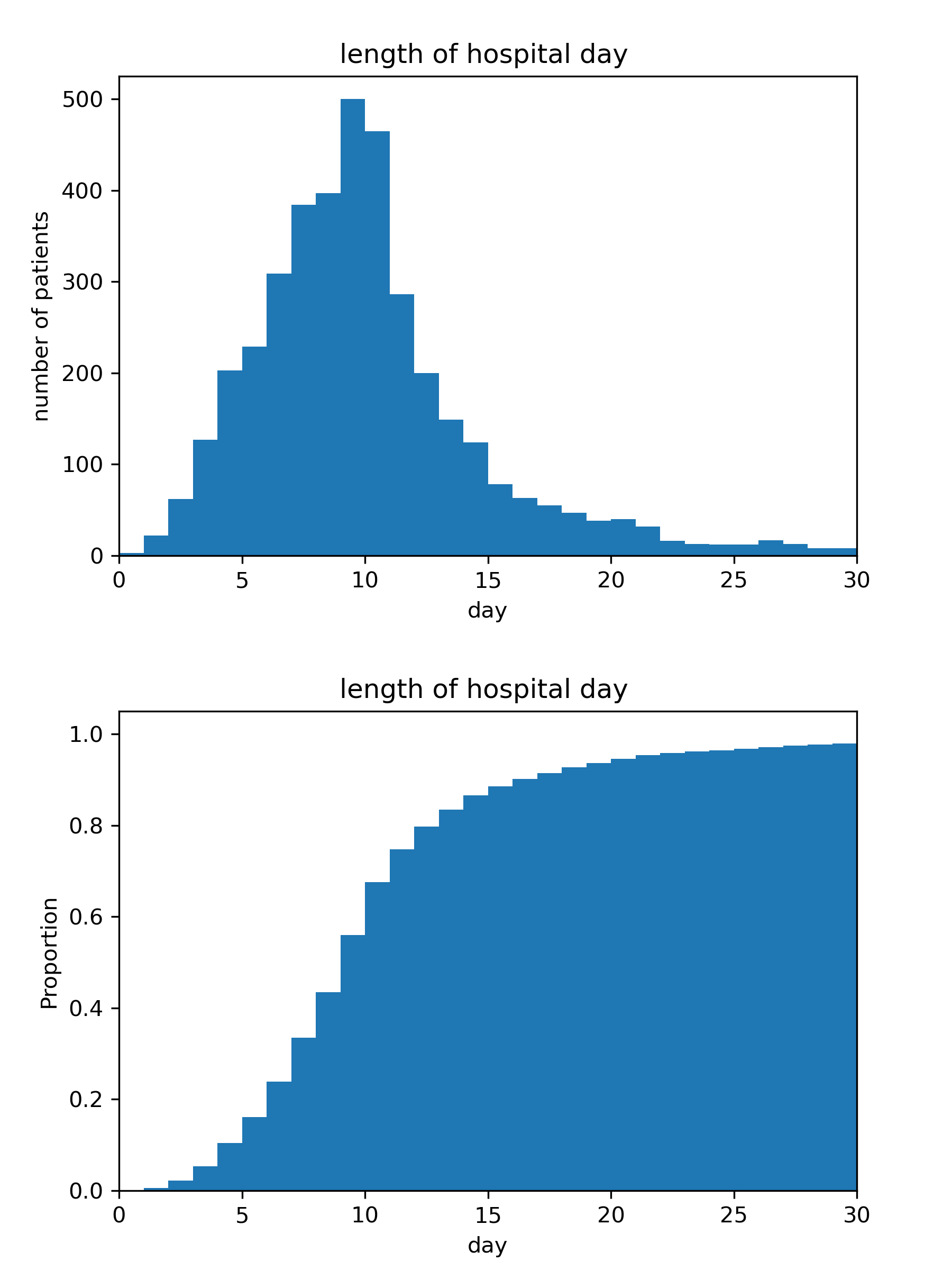

Supplement: S4 Fig — (TIF) [file pone.0294362.s005.tif]

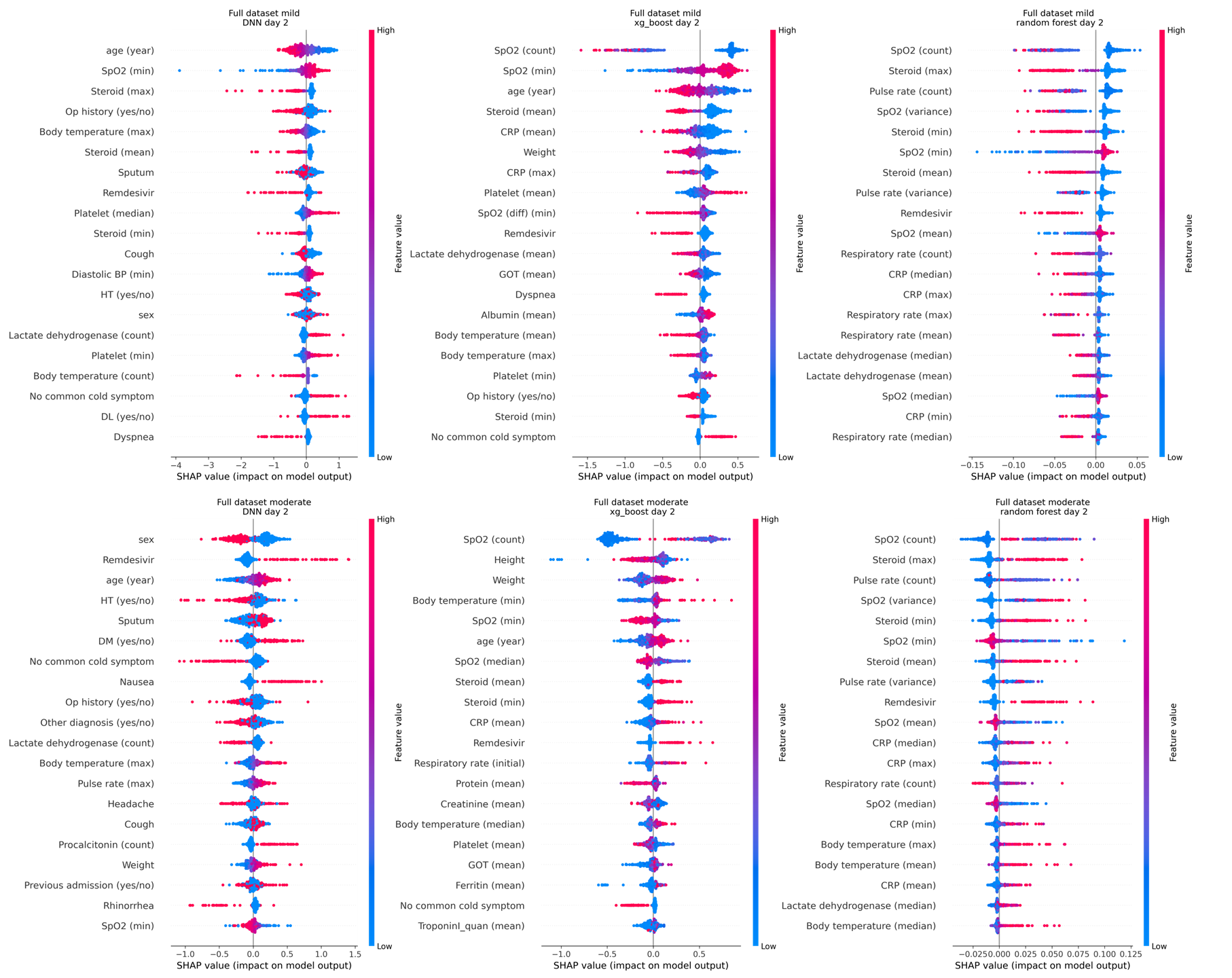

Supplement: S5 Fig — (TIF) [file pone.0294362.s006.tif]
