# Supplementary material for: In-hospital real-time prediction of COVID-19 severity regardless of disease phase using electronic health records
Source: PLoS One. 2024 Jan 25;19(1):e0294362. doi: 10.1371/journal.pone.0294362 (PMC10810421; doi:10.1371/journal.pone.0294362)
Supplement: S1 Table — (DOCX) [file pone.0294362.s007.docx]

S1 Table. Percentile and clipping values of continuous variables.

| Percentile | 0.05 | 0.5 | 1 | 99 | 99.5 | 99.95 |
| --- | --- | --- | --- | --- | --- | --- |
| CBC |  |  |  |  |  |  |
| WBC (×10^3^/uL) | 1 | 1.7 | **2** | **22.4** | 27.7 | 50 |
| Neutrophil (%) | 4.3 | **10.1** | 13.3 | **95.2** | 95.7 | 96.9 |
| Lymphocyte (%) | 0.7 | 1.7 | **2.2** | 75.7 | **79.5** | 86.2 |
| Eosinophil (%) | 0 | 0 | 0 | **11.4** | 14.4 | 21.1 |
| Hemoglobin (g/dL) | **5.4** | 6.9 | 7.2 | 16.7 | **17.1** | 18.4 |
| Platelet (×10^3^/uL) | 16 | 39.7 | **51** | **563** | 616 | 771.3 |
| Chemistry |  |  |  |  |  |  |
| Protein (g/dL) | 3.6 | 4.1 | **4.4** | **7.9** | 8 | 8.5 |
| Albumin (g/dL) | 1.5 | **1.9** | 2 | 4.9 | **5** | 5.4 |
| Alkaline phosphatase (IU/L) | 20 | **29** | 33 | 316 | **354.2** | 560.3 |
| Bilirubin (mg/dL) | 0.1 | 0.1 | **0.1** | 1.9 | 2.4 | **5.7** |
| Uric acid (mg/dL) | 0.7 | 1.2 | **1.4** | **9.5** | 10.4 | 14.7 |
| BUN (mg/dL) | 3 | 4 | **5** | **82.5** | 95 | 120 |
| Creatinine (mg/dL) | 0.2 | 0.2 | **0.2** | **5.7** | 9.1 | 19.9 |
| Total cholesterol (mg/dL) | 46 | 59 | **66.3** | **241** | 255 | 318.5 |
| Glomerular filtration rate (ml/min/1.73 m^2^) | 2.6 | 5.2 | **9.1** | **390.5** | 528.1 | 908.3 |
| Glucose (mg/dL) | 49 | **65** | 70 | **368** | 410 | 591.3 |
| AST (GOT) (IU/L) | 7 | 10 | **11** | 159.3 | **223.2** | 1070.9 |
| ALT (GPT) (IU/L) | 4 | 5 | **6** | 185 | **242.3** | 827.9 |
| γ-GT (IU/L) | 6 | 9 | **10** | **600** | 672 | 940.3 |
| Electrolyte |  |  |  |  |  |  |
| Sodium (mmol/L) | 114.9 | 123 | **125.1** | **148** | 151.3 | 168.4 |
| Potassium (mmol/L) | 2.5 | 2.9 | **3** | 5.5 | **5.8** | 6.9 |
| Inflammatory markers |  |  |  |  |  |  |
| Lactate dehydrogenase (IU/L) | 100 | 120 | **127.1** | **684.9** | 803.3 | 1484.1 |
| Creatine kinase (IU/L) | 9 | 16 | **18** | **1200** | 1965 | 25506.1 |
| C-reactive protein (mg/dL) | 0 | 0 | **0** | **22.2** | 25.6 | 34.9 |
| Ferritin (ng/mL) | 1 | 1 | **2.2** | **2004.5** | 2740 | 9979.7 |
| Procalcitonin (ng/mL) | 0 | 0 | **0** | **2.5** | 5.7 | 40.7 |
| Fibrinogen (mg/dL) | 106.7 | 179.7 | **204.7** | **828.7** | 880.9 | 999 |
| Troponin-I ((ng/mL)) | 2.3 | 2.3 | **2.3** | **877.8** | 4652.2 | 136993 |
| Coagulation related value |  |  |  |  |  |  |
| PT (INR) | 1 | 1 | **1** | 2 | 2.5 | **3.9** |
| PT (%) | 14.6 | **25.1** | 34.8 | **100** | 100 | 100 |
| PT (sec) | 9.7 | **10.3** | 10.4 | 21.6 | **26.7** | 41.2 |
| aPTT(Activated PTT) | 20.4 | 21.8 | **22.7** | **44.1** | 49.4 | 74.6 |
| D-dimer (ug/mL) | 0.2 | 0.2 | **0.2** | **28** | 34.1 | 35.2 |
| Vital signs |  |  |  |  |  |  |
| SBP (mmHg) | 38 | **68** | 97 | 196 | **208** | 256 |
| DBP (mmHg) | 22 | **36** | 54 | **106** | 111 | 160 |
| PR (/min) | 34 | **44** | 58 | 134 | 143 | **170** |
| RR (/min) | **6** | 11 | 18 | 38 | **42** | 59 |
| BT (℃) | 34.4 | **35.6** | 36.1 | **39** | 39.2 | 40 |
| SpO2 (%) | 53 | **77** | 93 | **100** | 100 | 100 |
