# Supplementary material for: In-hospital real-time prediction of COVID-19 severity regardless of disease phase using electronic health records
Source: PLoS One. 2024 Jan 25;19(1):e0294362. doi: 10.1371/journal.pone.0294362 (PMC10810421; doi:10.1371/journal.pone.0294362)
Supplement: S2 Table — (DOCX) [file pone.0294362.s008.docx]

S2 Table. Symptom presentation of COVID-19 patients during hospital admission

| Type of symptoms | Number of patients (%) |
| --- | --- |
| General symptoms |  |
| Myalgia | 989/3996 (24.75%) |
| Anorexia | 143/3996 (3.58%) |
| Headache | 1933/3996 (48.37%) |
| Febrile sense | 653/3996 (16.34%) |
| Gastrointestinal symptoms |  |
| Constipation | 97/3996 (2.43%) |
| Diarrhea | 1292/3996 (32.33%) |
| Dyspepsia | 380/3996 (9.51%) |
| Nausea | 1247/3996 (31.21%) |
| Vomiting | 374/3996 (9.36%) |
| Respiratory symptoms |  |
| Chest pain | 292/3996 (7.31%) |
| Cough | 3173/3996 (79.4%) |
| Dyspnea | 1125/3996 (28.15%) |
| Rhinorrhea | 671/3996 (16.79%) |
| Sore throat | 297/3996 (7.43%) |
| Sputum | 2748/3996 (68.77%) |

During admission, patients recorded their symptoms, and the proportion of the positive symptoms was described. If symptoms appear for more than one day, the symptoms are considered positive.
