# Supplementary material for: In-hospital real-time prediction of COVID-19 severity regardless of disease phase using electronic health records
Source: PLoS One. 2024 Jan 25;19(1):e0294362. doi: 10.1371/journal.pone.0294362 (PMC10810421; doi:10.1371/journal.pone.0294362)
Supplement: S3 Table — (DOCX) [file pone.0294362.s009.docx]

S3 Table. Detailed model performance comparison.

|  | Model name | Accuracy | AUROC | AUPRC | Precision | Recall | F1-Score |
| --- | --- | --- | --- | --- | --- | --- | --- |
| Day 0 | Logistic regression | 89 [88.4-89.6] | 0.962 [0.959-0.966] | 0.872 [0.861-0.883] | 0.885 | 0.89 | 0.886 |
|  | DNN | 92.9 [92.3-93.4] | 0.972 [0.969-0.976] | 0.934 [0.926-0.943] | 0.928 | 0.929 | 0.928 |
|  | XG boost | 95.7 [95.3-96.1] | 0.991 [0.99-0.993] | 0.972 [0.967-0.977] | 0.956 | 0.957 | 0.956 |
|  | Random forest | 95.2 [0.966-0.861] | 0.992 [0.99-0.993] | 0.977 [0.973-0.98] | 0.951 | 0.952 | 0.951 |
| Day 1 | Logistic regression | 87.1 [86.4-87.7] | 0.948 [0.944-0.952] | 0.837 [0.826-0.849] | 0.861 | 0.871 | 0.863 |
|  | DNN | 91.9 [91.3-92.4] | 0.966 [0.962-0.97] | 0.923 [0.913-0.931] | 0.916 | 0.919 | 0.916 |
|  | XG boost | 94.4 [94-94.9] | 0.985 [0.982-94.9] | 0.958 [0.951-0.963] | 0.943 | 0.944 | 0.942 |
|  | Random forest | 94.4 [93.9-94.9] | 0.988 [0.987-0.99] | 0.969 [0.965-0.973] | 0.943 | 0.944 | 0.942 |
| Day 2 | Logistic regression | 86.2 [85.5-86.9] | 0.938 [0.934-0.942] | 0.808 [0.795-0.82] | 0.846 | 0.862 | 0.848 |
|  | DNN | 91.6 [91.1-92.2] | 0.962 [0.958-0.966] | 0.913 [0.904-0.921] | 0.914 | 0.916 | 0.913 |
|  | XG boost | 93.6 [93.1-94.1] | 0.979 [0.976-0.982] | 0.946 [0.939-0.953] | 0.934 | 0.936 | 0.933 |
|  | Random forest | 93.9 [93.4-94.4] | 0.985 [0.983-0.987] | 0.959 [0.953-0.964] | 0.938 | 0.939 | 0.935 |
