# Supplementary material for: In-hospital real-time prediction of COVID-19 severity regardless of disease phase using electronic health records
Source: PLoS One. 2024 Jan 25;19(1):e0294362. doi: 10.1371/journal.pone.0294362 (PMC10810421; doi:10.1371/journal.pone.0294362)
Supplement: Attachment — Submitted filename: rebuttal_letter_plos_one1024.docx [file pone.0294362.s011.docx]

24 October 2023

To Academic Editor

Plos one

Dear Editor:

Thank you for the opportunity to revise and resubmit our manuscript entitled "In-hospital real-time prediction of COVID-19 severity regardless of disease phase using electronic health records" to PLOS ONE. We appreciate the reviewers' thoughtful and constructive feedback, which has helped strengthen our work.

Responding to Reviewer #4, we recognize their concerns about potential discrepancies between model predictions and observed clinical status, as well as risks of overfitting. We acknowledge these valid points that warrant consideration in the next phase of model development and prospective implementation. We hope to validate our model in real-world practice through a prospective study, where we can further evaluate clinical issues like overfitting and prediction disparities.

The reviewer's perspectives have meaningfully improved our manuscript. We believe it now meets PLOS ONE's publication criteria and look forward to your decision. Please contact me with any additional questions.

Sincerely,

Ji Bong Jeong

Department of Internal Medicine, Seoul National University College of Medicine, Seoul, Korea, Division of Gastroenterology, Department of Internal Medicine, Seoul Metropolitan Government Seoul National University Boramae Medical Center, 20, Boramae‐ro 5‐gil, Dongjak‐gu, Seoul, 07061, Republic of Korea.

Email: jibjeong@snu.ac.kr

Phone: +82-2-870-2222

Fax: +82-2-870-3861

Review Comments to the Author

Reviewer #3: The authors have addressed my comments. I have no further concerns. I think the authors did a good job addressing my comments

- **Thank you for your positive feedback and acknowledgment of our efforts in addressing your comments. We greatly appreciate your constructive insights throughout the review process.**

Reviewer #4: The article discusses the development and evaluation of machine learning and deep learning models to predict the daily severity of COVID-19 in patients at a dedicated hospital. The goal is to forecast the severity of the condition up to 2 days in advance to help manage healthcare resources efficiently. The study uses a dataset of COVID-19 patients from a specific medical center and assesses various models for their predictive accuracy.
The key findings and points in the article include:
- The importance of predicting the daily severity of COVID-19 to facilitate proactive resource allocation, such as respiratory devices.
- The comparison of different types of prediction models, including non-temporal (logistic regression, random forest, gradient boost) and temporal models (transformer).
- The use of patient data, including demographics, symptoms, laboratory tests, and vital signs as input features for prediction.
- The performance of the models in predicting severity for different time horizons (day 0, day 1, and day 2), with the random forest model outperforming others for predicting day 0 severity.
- The importance of feature selection and model interpretability using the SHAP method, with differences in feature importance between models.
- The discussion of model generalizability to other hospitals and the potential limitations of the study, including data availability and clinical variations.
In conclusion, the study presents a machine learning model, particularly the random forest model, as a valuable tool for predicting COVID-19 severity, aiding healthcare resource allocation, and offering insights into patient outcomes. The hierarchical transformer model also demonstrated good performance for certain periods of hospital admission. Further validation and research are needed to confirm the findings and adapt the model for use in different healthcare settings.

Comments that should be addressed by the authors:

**1. Early identification of patients who are on the path to recovery could provide a justification for reducing or discontinuing ventilatory support and potentially adjusting the timing of treatment interventions?**

- We value your insights. In our study, the primary goal was to proactively detect signs of patient deterioration and recovery. Although our model doesn't directly change patient trajectory upon early identification, its strength lies in the anticipation of potential deterioration. This early prediction becomes pivotal when considering resource allocation. On days where the model predicts fewer severe cases, receiving a call about a deteriorating patient might lead to more flexible allocation of resources, such as quickly assigning ventilatory support like HFNC. However, on days with a higher predicted count of at-risk patients, the same call might necessitate more judicious and possibly stringent resource allocation, ensuring that the most critical patients receive timely intervention. This nuanced approach to resource allocation, guided by our predictive model, aims to optimize patient care outcomes in varied scenarios.

**2. The utilization of an exceptionally large number of predictors raises concerns about potential overfitting of the model to the dataset.**

We acknowledge your concern regarding the potential for overfitting due to the extensive number of predictors. It's worth noting that our inputs predominantly consist of variables routinely collected during standard patient care, enhancing their potential for generalization across different hospitals. While many contemporary predictive models strive to incorporate as many inputs as possible, we are aware of the risks associated with overfitting. To mitigate this, we employed a methodological approach by partitioning the dataset into training, validation, and test sets—a standard practice that we have applied in our study. Furthermore, we recognize the potential for bias, especially given that some high-performing tree-based models may produce overfitted results by prioritizing hospital-dependent factors. As a proactive measure, we advocate for the use of deep learning models that exclude these potential biases (hospital-dependent elements), ensuring not only effective near-term outcome prediction but also facilitating model generalization across various hospitals.

**3. The algorithm predicts severity of disease within 2 days. How should a clinician interpret the results of the algorithm if the prediction is opposed to clinical evidence? For example, if a patient is at clinical stability and the algorithm predicts impeding severe disease, what kind of information can be deducted by the clinician to address the causes of impending aggravation of the disease?**

We recognize the significance of your concern. In time-series prediction models, including ours, there's an inherent challenge when the model's forecast diverges from the observed clinical status. This challenge isn't unique to our model but is a general consideration for all time-series prediction models. In our current study, our primary focus has been on forecasting potential risks and recovery. The question of how to act upon a prediction, especially when it appears to contradict clinical observations, is indeed the subsequent phase of our model's objectives. We understand that further prospective studies are warranted to assess the potential risks associated with such discrepancies. For the time being, our model has been developed with an emphasis on addressing the challenges of overcrowded hospitals, where clinicians are constantly grappling with decisions related to ICU admissions, HFNC assignments, and potential discontinuations. In such scenarios, our model could serve as a valuable tool for healthcare professionals navigating high-demand environments.
